# 1433. Factors Associated with Unsuccessful Treatment Outcomes among Patients with Rifampicin Resistant Tuberculosis - Tajikistan, 2015-2020

**DOI:** 10.1093/ofid/ofac492.1262

**Published:** 2022-12-15

**Authors:** Zulfiya Haybullo Tilloeva, Amidkhan S Sijotkhonov, Sanam Zikriyarova, Bobochon Pirmahmadzoda, Sevak Alaverdyan, Dilyara Nabirova, Roberta Horth, Navruz Dzhafarov

**Affiliations:** City Desinfection station, Dushanbe, Republic of Tajikistan, Dushanbe, Dushanbe, Tajikistan; Center for State Sanitary and Epidemiological Surveillance, Dushanbe, Republic of Tajikistan, Dushanbe, Dushanbe, Tajikistan; Kazakh National Medical University named after S.D. Asfendiyarov;, Almaty, Almaty, Kazakhstan; City Center for Protection of the Population against Tuberculosis, Dushanbe, Republic of Tajikistan, Dushanbe, Dushanbe, Tajikistan; American University of Armenia, Dushanbe, Yerevan, Armenia; US Centers for Disease Control and Prevention, Regional Office of Central Asia, Almaty, Kazakhstan, Almaty, Almaty, Kazakhstan; US Centers for Disease Control and Prevention, Dulles, Virginia; Ministry of Health and Social Protection of the Population of the Republic of Tajikistan, Dushanbe, Dushanbe, Tajikistan

## Abstract

**Background:**

Tajikistan has a high burden of multidrug resistant TB, including rifampicin-resistant TB (RR-TB). Treatment options are more limited and include standard treatment (T1), bedaquiline/delamanid contained individual treatment (T2), short treatment (T3), modified short treatment (T4), and bedaquiline/pretomanid/linezolid treatment (T5). To inform national treatment plans, we assessed trends in RR-TB and determined factors associated with unsuccessful treatment.

**Methods:**

We conducted a retrospective cohort study in Dushanbe, Tajikistan, from 2015 to 2020 using patient records. Patients had successful treatment outcomes if they completed treatment or were cured, and unsuccessful if they had treatment failure, were lost-to-follow-up, or died. We report relative risk (RR) and 95% confidence interval from multivariable Poisson regression.

**Results:**

From 2015 to 2020, there was a decrease in incidence of TB (68.6 to 47.2 per 100,000) and RR-TB (10.6 to 7.2) with a 33% drop 2019 to 2020. Proportion of unsuccessful outcomes also decreased (30% to 15% 2015-2020). Of 522 patients, 515 had RR-TB. Of these, 39% received T1, 43% T2, 15% T3, 2% T4, and 0.4% T5 (Table 1). Overall, 23% had unsuccessful treatment outcomes. Patients on T1 had higher unsuccessful outcome compared to those on T2 - T5 (31% vs 19%, 14%, 0% and 0%, respectively). Unsuccessful outcomes were associated with combined isoniazid, rifampin and streptomycin resistance, alcohol and drug use. For patients receiving T1, unsuccessful outcome was associated with unemployment (RR: 2.7; 1.1-6.8), HIV (RR: 2.3, 1.5-3.4), hepatitis C (RR: 2.2, 1.4-3.4), and concomitant disease (RR: 2.1, 1.4-3.1). For patients receiving T2, risk of unsuccessful outcome was lower for females (RR: 0.4, 0.2-0.7) (Table 2).

**Figure:**
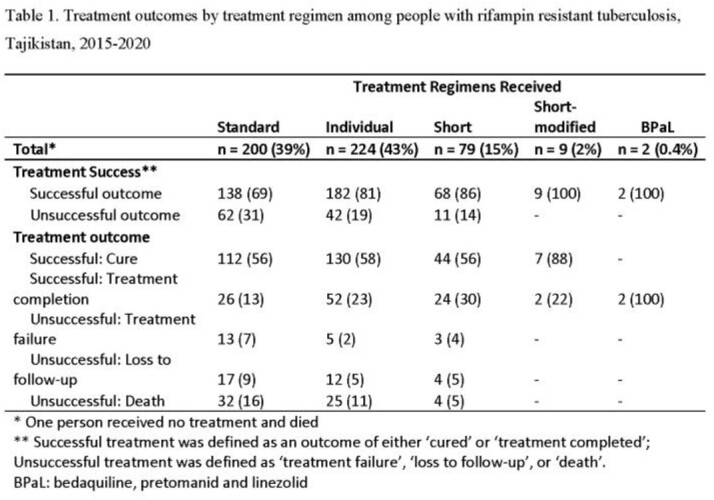

**Figure:**
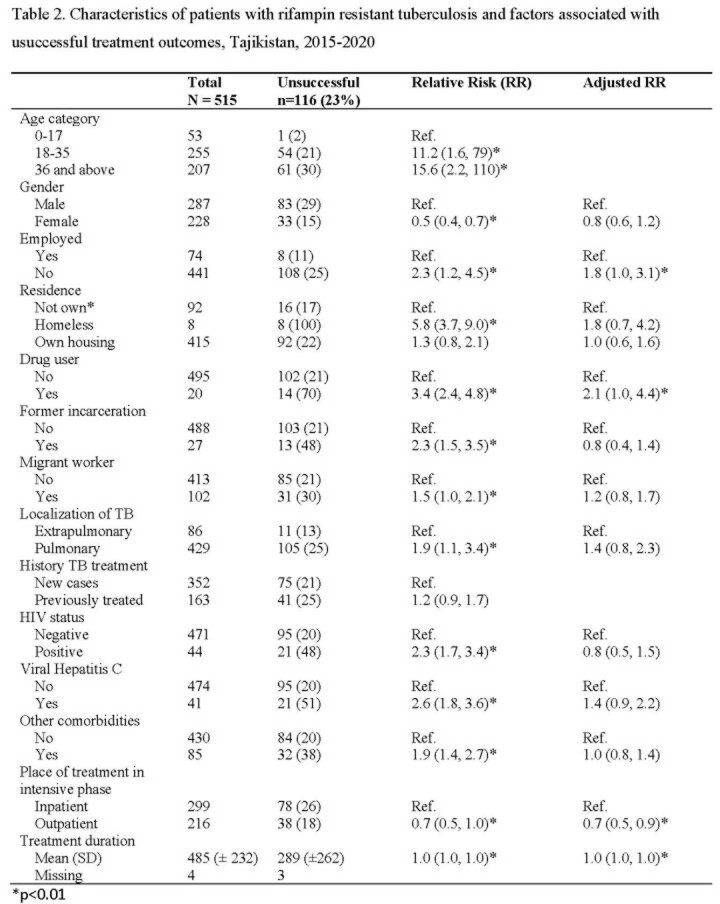

**Conclusion:**

Decrease in incidence is promising. Results highlight the need to transition RR-TB patients to non-standard treatment. Closer follow-up of patients with combined resistance, alcohol or drug use, HIV, hepatitis C, and concomitant diseases may contribute to successful outcome.

**Disclosures:**

**All Authors**: No reported disclosures.

